# Does microcycle length influence the external and internal load in professional female soccer players?

**DOI:** 10.5114/biolsport.2025.144408

**Published:** 2024-10-25

**Authors:** Manuel Posse-Álvarez, David Solleiro-Duran, Miguel Lorenzo-Martínez, Eliseo Iglesias-Soler, José M. Oliva-Lozano, Alexis Padrón-Cabo

**Affiliations:** 1 University of A Coruna, Department of Physical and Sports Education, Faculty of Sport Sciences and Physical Education, A Coruña, Spain; 2 High Performance Department, Real Club Deportivo de La Coruña, A Coruña, Spain; 3 High Performance Department, Olympique de Marseille, Marseille, France; 4 University of Vigo, Faculty of Education and Sport Sciences, Pontevedra, Spain; 5 Performance and Health Group, Department of Physical Education and Sport, Faculty of Sports Sciences and Physical Education, University of A Coruna, A Coruña, Spain; 6 United States Soccer Federation. Chicago, IL, United States

**Keywords:** Football, Women, GPS, Physical demands, Workload monitoring

## Abstract

This study aimed to analyze the effects of microcycle length on external and internal load during training sessions and official matches in a professional female soccer team. A total of 32 microcycles were monitored for 20 outfield players using a portable 10 Hz GPS device. Microcycles were categorized by length as short (5–6 days), regular (7 days), or long (8–9 days). Training load during the microcycles was analyzed relative to the days before or after the match day (MD), considering the following sessions: MD+1, MD-4, MD-3, MD-2, and MD-1. The external load was assessed according to total distance (TD), high-intensity running distance (HIR), sprint distance (SPD), and number of high-intensity accelerations (ACC) and decelerations (DEC). Internal load was measured using the rating of perceived exertion (RPE) and session RPE (s-RPE). The results of linear mixed model analyses showed that TD (d = 1.24–1.35), ACC (d = 0.64–0.66), and DEC (d = 0.90–1.04) were significantly higher on MD+1 in short microcycles compared to regular and long microcycles. On MD-4, large differences were observed between long and short microcycles for TD (d = 1.60), ACC (d = 1.28), DEC (d = 1.28), RPE (d = 1.25), and s-RPE (d = 1.61). Similarly, MD-3 of long microcycles showed significantly higher TD (d = 1.25–1.32), HIR (d = 1.18–1.32), and SPD (d = 0.84–1.68) compared to regular and short microcycles. On MD-2, the highest workload was observed in short microcycles, especially for SPD (d = 1.15–1.34). Overall, this study demonstrated that the external and internal load of training sessions vary depending on the length of the microcycles in professional female soccer.

## INTRODUCTION

In the context of team sports, the player workload has been considered as the input variable that can be manipulated to adjust the dose-response relationship [1]. Player workload can be organized as external load, which includes the physical work performed by the individual, and internal load, which refers to the psychophysiological response to these stimuli [2]. The use of Global Positioning Systems (GPS) technology provides valuable information on external load in terms of the volume and intensity of each training session and match [3]. Previous research has shown that over- or under-exposure to some external load metrics, such as high-speed running and sprint distance, may increase the risk of non-contact injuries [4, 5]. Similarly, higher accumulated total distance and player load were associated with injury in female soccer players [6]. The internal load was also shown to be effective in predicting changes in performance and injury risk in professional soccer [7, 8]. Consequently, adequate monitoring and management of training and match loads has been considered a crucial issue to optimize microcycle programming, prevent high fatigue levels and reduce injury risk in professional soccer [6, 9–11].

In recent years, female’s soccer has experienced an increase in competitiveness, resulting in significantly greater match and training demands [12, 13]. Despite this growth, there is limited research on training load quantification and programming strategies to better understand weekly planning and content selection [9]. In this regard, training load distribution in men’s soccer has been extensively studied [11, 14, 15]. However, these findings cannot be directly applied to female players due to potential differences in physiological status, physical fitness, and training background [16–19]. Specifically, professional female soccer players exhibited differences in peak match demands in total distance (~10%), very high-speed running (~16%), sprint distance (~55%), and the sum of accelerations and decelerations (~81%) compared to their male counterparts [20–22]. Recent studies have analyzed external load and internal load dynamics during the competitive microcycle according to the days with respect to the previous or next match day (MD) in professional female soccer teams [13, 23, 24]. Notably, workload was higher in the middle of the week (i.e., MD-4 and MD-3) than in the training sessions immediately before (i.e., MD-1) or after (i.e., MD+1) the match [13, 23, 24], similar to previous findings in professional male soccer players [11, 15].

During a competitive microcycle, the workload could be influenced by different contextual factors such as the length of the microcycle [25, 26], match schedule [27, 28], or season period [29]. Specifically, recent studies have highlighted the potential influence of the length of the microcycle in training load programming in men’s soccer [3, 30–32]. For instance, Oliva-Lozano et al. [26] observed that volume- and intensity-related variables were increased during longer microcycles (i.e., 8-day or 9-day microcycles) in professional male soccer players. Furthermore, Gualtieri et al. [33] established that practitioners adjusted microcycle programming based on the specific lengths of congested fixture microcycle (i.e., 3-, 4-, 5- days), reducing muscle impact (i.e., accelerations and decelerations) and using 5-day microcycle as recovery opportunity. To date, no previous research has analyzed the effect of the microcycle length on weekly workload in terms of volume and intensity in female soccer players. However, understanding the effect of the microcycle length seems to be crucial for coaches and sport scientists when it comes to training load management purposes throughout the competitive period.

**TABLE 1:** Training contents based on the length of microcycle.

Type of microcycle	Day	Description
Short Microcycle	MD+1	Regeneration exercises and low-impact activities at the gym for the group of players who played more than 60 minutes. The players who played less that 60 minutes: rondos, high intensity circuits and small sided-games
	MD-4	Preventive strength, possessions and positional games
	MD-3	Strength day, medium position games, small sided-games, transition drills, medium and large sided-games and 11 vs 11 matches
	MD-2	Control and passing tasks, tactical drills, small-sided games, medium and large sided-games and 11 vs 11 matches
	MD-1	Preventive strength, activation drills, rondos, shooting drills and offensive and defensive set pieces
	MD	Match day

Regular Microcycle	MD+1	Regeneration exercises and low-impact activities at the gym for the group of players who played more than 60 minutes. The players who played less that 60 minutes: rondos, high intensity circuits and small sided-games
	MD+2	Resting day
	MD-4	Strength day, circuits, possessions and small sided-games
	MD-3	Control and passing tasks, medium position games, medium and large sided-games and 11 vs 11 matches
	MD-2	Functional strength, rondos, tactical drills, small-sided games
	MD-1	Preventive strength, activation drills, rondos, shooting drills and offensive and defensive set pieces
	MD	Match day

Large Microcycle	MD+1	Regeneration exercises and low-impact activities at the gym for the group of players who played more than 60 minutes. The players who played less that 60 minutes: rondos, high intensity circuits and small sided-games
	MD+2	Resting day
	MD-4	Strength day, circuits, possessions and small sided-games
	MD-3	Control and passing tasks, medium position games, medium and large sided-games and 11 vs 11 matches
	MD-2	Functional strength, rondos, tactical drills, small-sided games
	MD-1	Preventive strength, activation drills, rondos, shooting drills and offensive and defensive set pieces
	MD	Match day

MD: match day; MD+1: 1 day after match; MD-4: 4 days before match; MD-3: 3 days before match; MD-2: 2 days before match; MD-1: 1 day before match.

Therefore, bearing in mind the aforementioned considerations, the aim of the current study was to analyze the influence of microcycle length on external and internal load in a professional female soccer team. Based on previous research conducted in male soccer [26], it could be hypothesized that the load of training sessions will depend on the length of microcycles, with longer microcycles showing a higher training load on the central days (i.e., MD-4 and MD-3). The findings of the current study may inform the practices of coaches and strength and conditioning specialists engaged in the training of female soccer teams, enabling them to enhance load management and microcycle programming to account for the varying lengths of microcycles.

## MATERIALS AND METHODS

### Experimental approach to the problem

A retrospective longitudinal observational research design was conducted on a professional female soccer team in the second Spanish division during the 2022–2023 season. According to Akenhead et al. [34], the data collected were classified in relation to the MD: 1 day before the match (MD-1), 2 days before the match (MD-2), 3 days before the match (MD-3), 4 days before the match (MD-4), and 1 day after the match (MD+1). In the MD+1 session, only the training session of players who have played less than 60 minutes was considered. In order to compare the training load of microcycles with different durations, the MD-5 or MD+2 were excluded, as these sessions are not part of the common structure of all types of microcycles. In addition, each microcycle was classified according to the average length of the microcycle, which was determined to be an average of 7 days [26]. Specifically, the microcycles were categorized as: short microcycles (SM; 5–6 days, n = 8), regular microcycles (RM; 7 days, n = 13), and long microcycles (LM; 8–9 days, n = 11). Table 1 presents the training contents of each microcycle day based on the length of the microcycle. Finally, a total of 1684 observations from a total of 32 microcycles were recorded for subsequent statistical analysis.

### Participants

A total of 20 professional female soccer players participated in this study (age: 23.7 ± 3.3 years; height: 166.4 ± 8.0 cm; body mass: 58.6 ± 6.3 kg). Based on their positional role, the players included were distributed as follows: central defenders (n = 4), fullbacks (n = 4), midfielders (n = 5), wide midfielders (n = 4), and forwards (n = 3). Specifically, the goalkeepers were excluded from the data analysis due to their different external load demands compared to outfield players. Additionally, only those players who completed the full match or training session were included in the statistical analysis. All participants had a minimum of one year and a maximum of 12 years of experience at the professional soccer level. Players regularly trained 5 times per week, and training sessions ranged from 45 to 90 min (average duration 64.7 ± 19.6 min) depending on the day of the microcycle. As data used in this study were collected as part of routine player monitoring, no ethics committee approval was required [35]. All players and the team provided informed consent to the use of data for the purposes of this research.

### Procedures

During the observation period, the external load of each training session and match was recorded using a portable 10 Hz GPS device (Yoomedoo, Palma de Mallorca, Balearic Islands, Spain). According to Scott et al. [36], GPS devices with a sampling rate of 10 Hz are the most valid and reliable for external load analysis in team sports. Furthermore, based on the technical specifications, this device provides high levels of validity and accuracy due to its horizontal position accuracy (i.e., 30 cm under optimal conditions) and velocity accuracy (i.e., 0.03 m/s). To avoid inter-unit variability errors, all players used the same GPS device during training sessions and matches. All GPS units were activated 15 minutes before each training session or match in an open area to acquire at least 6 satellites to avoid weak connections, ensure data quality, and increase the reliability of data collection [37, 38]. Each player wore a sports waistcoat with a pocket for the GPS device provided by the company. Upon completion of each training session and match, data were exported and adjusted using the manufacturer’s software for the data collection.

External load metrics were analyzed in terms of volume (e.g., absolute distance covered in meters or number of actions) and intensity (e.g., distance covered or number of actions relative to the duration of a training session or match). In line with previous research [39, 40], the following kinematic variables were calculated: total distance (TD), high-intensity running distance (HIR; 19–23 km · h^−1^), and sprint distance (SPD; > 23 km · h^−1^). The following mechanical metrics were quantified: number of high-intensity accelerations (ACC; > 3 m · s^−2^) and number of high-intensity decelerations (DEC; > -3 m · s^−2^). All these variables were also analyzed in relative terms (“R” before the distance category). In addition, the maximum speed reached by the players in each session was recorded.

The 0–10 Foster’s Rating of Perceived Exertion (RPE) was used to measure and assess the internal workload reported by each player [41]. After training sessions or matches, all participants individually recorded their RPE 30 minutes later to avoid potential bias [42]. The RPE scale was already familiar to the players as they used it regularly during their training routine. Furthermore, sRPE was calculated by multiplying the RPE score of each player by the duration of the training session.

### Statistical Analyses

Descriptive statistics for each variable were presented as mean and standard deviation (mean ± SD). All statistical analyses were performed using the statistical software R version 4.2.3 [43]. A linear mixed model was adjusted using the R package “*lm4* ” [44] to analyze the differences in external load variables according to microcycle length (i.e., short, regular, and long microcycles) and microcycle session (i.e., MD+1, MD-4, MD-3, MD-2, MD-1, MD). This hierarchical mixed model was applied to deal with different numbers of observations per player and repeated measures data [45]. Specifically, the microcycle length and microcycle sessions were included as fixed factors in the model. Additionally, player identity was included as a random effect to account for repeated measures. For each external load variable (*y*), the model structure was adjusted as follows:

y=Microcycle Length×Session+(1|Player Identity)

For each linear mixed model, the homogeneity and normal distribution of the residuals were examined, and all models accomplished both assumptions. Likewise, Bonferroni’s post-hoc test was used to analyze the differences in external load according to the fixed factors. These pairwise comparisons were performed using the R package “*emmeans* ” [46]. To determine a standardized effect size (ES), Cohen’s *d* was calculated using the following formula: *d* = (M_2_ – M_1_)/SD_pooled_. Based on Hopkins et al. [47], Cohen’s *d* was interpreted using the following thresholds: trivial (0.0–0.2), small (0.2–0.6), moderate (0.6–1.2), large (1.2–2.0), and very large (> 2.0). For all statistical analyses, the alpha level was set at *p* ≤ 0.05.

## RESULTS

### Effects of microcycle length on absolute workload metrics of training and match day

Table 2 shows the differences in absolute workload values (i.e., volume variables) according to microcycle length and session. Specifically, the SM had a significantly longer match duration than the LM (*d* = 0.47, small). In terms of match RPE and sRPE, both SM (*d* = 0.58 and 0.56, small) and LM (*d* = 0.38 and 0.39, small) displayed significantly lower values compared to RM.

**TABLE 2:** Differences (mean ± SD) in the distance covered in different speed zones according to the length of microcycle.

Variable	Microcycle Length	MD+1	MD-4	MD-3	MD-2	MD-1	MD
Duration (min)	Short Microcycle	57.32 ± 14.68	61.07 ± 10.01	73.21 ± 15.20	63.86 ± 5.63	50.52 ± 15.28	94.05 ± 1.45
	Regular Microcycle	41.77 ± 3.57^*^	62.04 ± 13.56	64.22 ± 8.15^*^	59.91 ± 12.07^$^	56.85 ± 9.75^$^	93.70 ± 1.11
	Long Microcycle	42.86 ± 6.26^*^	77.14 ± 3.92^*#^	85.93 ± 9.75^*#^	67.22 ± 8.10^$#^	41.55 ± 8.71^*#^	93.52 ± 0.88^$^

TD (m)	Short Microcycle	3700.29 ± 812.64	3630.69 ± 953.92	5039.02 ± 1096.30	4415.88 ± 888.96	2398.43 ± 699.54	9380.00 ± 762.08
	Regular Microcycle	2865.91 ± 350.45^*^	3902.07 ± 874.49	5150.24 ± 805.49	3777.71 ± 816.72^*^	2117.86 ± 321.20	9273.13 ± 814.75
	Long Microcycle	2806.91 ± 508.58^*^	4960.69 ± 808.40^*#^	6434.66 ± 1131.84^*#^	4157.73 ± 945.49^*#^	1904.00 ± 510.66^*^	9375.44 ± 805.81

HIR (m)	Short Microcycle	54.41 ± 36.94	88.27 ± 157.39	108.80 ± 115.54	98.87 ± 66.13	23.48 ± 28.99	251.00 ± 108.81
	Regular Microcycle	66.81 ± 35.90	51.06 ± 48.86	144.54 ± 122.36^*^	69.36 ± 47.76	13.21 ± 23.42	265.52 ± 97.21
	Long Microcycle	83.81 ± 89.65	105.86 ± 115.14^#^	344.15 ± 206.87^*#^	77.24 ± 62.65	10.38 ± 17.61	267.35 ± 95.70

SPD (m)	Short Microcycle	22.05 ± 23.84	2.75 ± 9.21	6.84 ± 9.93	57.83 ± 53.31	3.56 ± 9.93	63.00 ± 47.51
	Regular Microcycle	15.91 ± 19.91	5.06 ± 10.85	41.75 ± 57.65^*^	11.22 ± 17.36^*^	1.78 ± 5.48	77.76 ± 52.51
	Long Microcycle	10.24 ± 18.80	10.82 ± 19.52	93.44 ± 64.72^*#^	16.11 ± 23.24^*^	0.62 ± 4.60	74.11 ± 50.59

ACC (count)	Short Microcycle	19.52 ± 10.45	10.34 ± 7.12	22.03 ± 12.38	12.51 ± 6.30	7.73 ± 4.74	26.42 ± 6.68
	Regular Microcycle	13.13 ± 8.41^*^	20.04 ± 10.46^*^	15.45 ± 7.59^*^	11.16 ± 6.07	4.86 ± 3.70	24.86 ± 7.63
	Long Microcycle	14.02 ± 6.82^*^	21.84 ± 9.34^*^	19.92 ± 8.67^#^	14.08 ± 7.05^#^	7.34 ± 4.33	26.31 ± 6.89

DEC (count)	Short Microcycle	21.59 ± 9.49	11.13 ± 7.33	24.28 ± 12.02	15.66 ± 7.53	6.83 ± 4.45	35.45 ± 10.27
	Regular Microcycle	14.00 ± 6.36^*^	21.32 ± 10.82^*^	18.10 ± 7.74^*^	12.93 ± 7.53^$^	4.75 ± 3.63	33.73 ± 12.00
	Long Microcycle	13.11 ± 6.92^*^	23.59 ± 10.15^*†^	21.12 ± 9.38^*†^	15.68 ± 8.87^#^	5.41 ± 3.48	35.84 ± 9.71

RPE (AU)	Short Microcycle	5.11 ± 1.39	3.93 ± 2.12	5.81 ± 1.20	4.78 ± 1.18	2.08 ± 0.81	7.47 ± 1.60
	Regular Microcycle	5.44 ± 0.52	5.06 ± 1.35^*^	5.68 ± 1.24	4.03 ± 1.17^*^	2.17 ± 0.66	8.32 ± 1.39^*^
	Long Microcycle	6.11 ± 0.89^*^	5.61 ± 1.14^*#^	6.34 ± 1.32^*#^	4.13 ± 1.47^*^	1.91 ± 0.73	7.79 ± 1.43^†^

sRPE (AU)	Short Microcycle	288.97 ± 95.11	255.51 ± 178.49	433.03 ± 156.53	302.11 ± 78.81	107.52 ± 56.57	702.70 ± 149.84
	Regular Microcycle	212.33 ± 20.55	322.27 ± 121.61^$^	365.96 ± 100.28^*^	246.84 ± 105.83^*^	123.00 ± 39.85	780.85 ± 134.15^*^
	Long Microcycle	265.14 ± 65.61	433.10 ± 91.55^*#^	549.40 ± 144.81^*#^	282.58 ± 115.94^†^	81.03 ± 37.48	729.00 ± 134.34^†^

Max. Speed (km/h)	Short Microcycle	24.45 ± 2.40	20.49 ± 2.17	23.24 ± 1.53	24.76 ± 2.48	20.99 ± 2.28	25.66 ± 1.33
	Regular Microcycle	23.47 ± 2.00	22.02 ± 2.29^*^	24.55 ± 1.83^*^	23.32 ± 2.02^*^	19.47 ± 2.81^*^	26.21 ± 1.72
	Long Microcycle	22.19 ± 2.42^*^†	22.50 ± 2.04^*^	25.84 ± 1.62^*#^	23.41 ± 2.13^*^	19.41 ± 2.18^*^	26.35 ± 1.52

Abbreviations: TD: total distance; HIR: high-intensity running; SPD: sprint distance; ACC: high-intensity accelerations (> 3 m/s^2^); DEC: high-intensity accelerations (> 3 m/s^2^); RPE: rating of perceived exertion; sRPE: session rating of perceived exertion; MD: match day; MD+1: 1 day after match; MD-4: 4 days before match; MD-3: 3 days before match; MD-2: 2 days before match; MD-1: 1 day before match;

* Significant differences (p < 0.01) with short microcycle.

# Significant differences (p < 0.01) with regular microcycle.

† Significant differences (p < 0.05) with short microcycle.

$ Significant differences (p < 0.05) with regular microcycle.

Regarding MD+1, the SM showed significantly higher TD (*d* = 1.24 and 1.35, large), ACC (*d* = 0.66 and 0.64, moderate), and DEC (*d* = 0.90 and 1.04, moderate) than RM and LM. The SM (*d* = 0.94, moderate) and RM (*d* = 0.56, small) also showed significantly greater maximal speed than LM. In addition, SM showed significantly lower RPE values than LM (*d* = 0.88, moderate) and longer training session duration compared to RM and LM (*d* = 1.33 and 1.33, large).

For MD-4, the LM displayed significantly higher TD (*d* = 1.26, large), HIR (*d* = 0.62, moderate), DEC (*d* = 0.22, small), and training session duration (*d* = 1.50, large) than RM. Compared to SM, LM also showed significantly greater TD (*d* = 1.60, large) and training session duration (*d* = 2.98, very large). Both LM and RM showed significantly greater ACC (*d* = 1.28 and 0.97, moderate), DEC (*d* = 1.28 and 0.99, large and moderate), and maximal speed (*d* = 0.98 and 0.67, moderate) than SM. In terms of RPE and sRPE, LM exhibited significantly higher values than SM (*d* = 1.25 and 1.61, large) and RM (*d* = 0.44 and 1.03, small and moderate), while RM showed significantly higher values than SM (*d* = 0.75 and 0.51, moderate and small).

Concerning MD-3, the LM showed significantly higher TD (*d* = 1.25 and 1.32, large), HIR (*d* = 1.32 and 1.18, large and moderate), SPD (*d* = 1.68 and 0.84, large and moderate), maximal speed (*d* = 1.64 and 0.74, large and moderate), and training session duration (*d* = 1.05 and 2.92, moderate and very large) than SM and RM. Likewise, RM displayed significantly higher HIR (*d* = 0.30, small), SPD (*d* = 0.75, moderate), and maximal speed (*d* = 0.76, moderate) than SM. DEC were significantly greater in SM than in RM (*d* = 0.65, moderate) and LM (*d* = 0.30, small), and also higher in LM than in RM (*d* = 0.35, small). In addition, RM had significantly lower ACC than SM (*d* = 0.69, moderate) and LM (*d* = 0.55, small). For RPE and sRPE, LM presented higher values compared to SM (*d* = 0.42 and 0.78, small and moderate) and RM (*d* = 0.52 and 1.48, small and large), with SM also showing higher sRPE than RM (*d* = 0.54, small).

With respect to MD-2, the SM showed significantly greater SPD (*d* = 1.34 and 1.15, large and moderate), maximal speed (*d* = 0.66 and 0.60, moderate), and RPE (*d* = 0.64 and 0.47, moderate and small) compared to RM and LM. Likewise, RM had significantly lower TD (*d* = 0.76 and 0.43, moderate and small), DEC (*d* = 0.36 and 0.34, small), training session duration (*d* = 0.39 and 0.72, small and moderate), and sRPE (*d* = 0.57 and 0.32, small) than SM and LM, and also lower ACC than LM (d = 0.44, small). In addition, the SM had significantly higher TD (*d* = 0.29, small) but lower training session duration (*d* = 0.46, small) than LM.

Finally, on MD-1, the SM exhibited significantly higher maximal speed values than RM and LM (*d* = 0.64 and 0.71, moderate), and also higher TD than LM (*d* = 0.79, moderate). The RM had significantly longer training session durations than SM (*d* = 0.44, small) and LM (*d* = 1.70, large), and SM also had longer training session durations than LM (*d* = 0.69, moderate).

### Effects of microcycle length on relative workload load metrics of training and match day

Table 3 presents the relative workload (i.e., intensity variables) for locomotor and neuromuscular metrics according to microcycle length and session. For MD+1, the SM had significantly lower RHIR (*d* = 0.61, moderate) and RDEC (*d* = 0.55, small) than LM.

**TABLE 3:** Differences (mean ± SD) in the distance covered in different speed zones according to the length of microcycle.

Variable	Microcycle Length	MD+1	MD-4	MD-3	MD-2	MD-1	MD
RTD (m/min)	Short Microcycle	65.37 ± 8.19	58.95 ± 8.44	69.29 ± 9.89	68.87 ± 10.86	48.83 ± 10.60	99.81 ± 8.83
	Regular Microcycle	68.47 ± 4.26	64.07 ± 14.43*	80.66 ± 10.87*	63.37 ± 8.22*	37.74 ± 5.11*	98.96 ± 8.58
	Long Microcycle	65.41 ± 6.52	64.38 ± 10.74*	74.81 ± 10.10*^#^	61.48 ± 9.84*	45.82 ± 7.96^#^	100.24 ± 8.51

RHIR (m/min)	Short Microcycle	1.00 ± 0.69	1.22 ± 2.08	1.35 ± 1.14	1.56 ± 1.09	0.48 ± 0.62	2.67 ± 1.17
	Regular Microcycle	1.65 ± 0.99	0.79 ± 0.74	2.19 ± 1.60*	1.18 ± 0.79	0.21 ± 0.37	2.83 ± 1.03
	Long Microcycle	2.05 ± 2.21*	1.35 ± 1.44^#^	3.87 ± 2.13*^#^	1.16 ± 0.96^$^	0.24 ± 0.37	2.86 ± 1.02

RSPD (m/min)	Short Microcycle	0.34 ± 0.37	0.04 ± 0.12	0.09 ± 0.14	0.91 ± 0.89	0.06 ± 0.18	0.67 ± 0.50
	Regular Microcycle	0.40 ± 0.50	0.08 ± 0.18	0.63 ± 0.81*	0.19 ± 0.31*	0.02 ± 0.09	0.83 ± 0.56
	Long Microcycle	0.23 ± 0.40	0.14 ± 0.24	1.05 ± 0.69*^#^	0.23 ± 0.34*	0.01 ± 0.10	0.79 ± 0.54

RACC (count/min)	Short Microcycle	0.36 ± 0.20	0.16 ± 0.10	0.29 ± 0.14	0.19 ± 0.09	0.17 ± 0.12	0.28 ± 0.07
	Regular Microcycle	0.31 ± 0.17	0.32 ± 0.16*	0.24 ± 0.11*	0.19 ± 0.10	0.09 ± 0.08*	0.27 ± 0.08
	Long Microcycle	0.33 ± 0.16	0.28 ± 0.12*^†^	0.23 ± 0.09*	0.21 ± 0.10	0.18 ± 0.11^#^	0.28 ± 0.07

RDEC (count/min)	Short Microcycle	0.39 ± 0.18	0.18 ± 0.11	0.34 ± 0.13	0.24 ± 0.11	0.15 ± 0.10	0.38 ± 0.11
	Regular Microcycle	0.33 ± 0.13	0.34 ± 0.18*	0.28 ± 0.12*	0.21 ± 0.11	0.08 ± 0.06^$^	0.36 ± 0.13
	Long Microcycle	0.30 ± 0.15*	0.30 ± 0.13*^#^	0.24 ± 0.11*^#^	0.23 ± 0.12	0.14 ± 0.09	0.38 ± 0.10

Abbreviations: RTD: relative total distance; RHIR: relative high-intensity running distance; RSPD: relative sprint distance; RACC: relative high-intensity accelerations (> 3 m/s^2^); RDEC: relative high-intensity accelerations (> 3 m/s^2^); MD: match day; MD-5: 5 days before match; MD-4: 4 days before match; MD-3: 3 days before match; MD-2: 2 days before match; MD-1: 1 day before match;

* Significant differences (p < 0.01) with short microcycle.

# Significant differences (p < 0.01) with regular microcycle.

$ Significant differences (p < 0.05) with short microcycle.

† Significant differences (p < 0.05) with regular microcycle.

Regarding MD-4, the SM showed lower RTD (*d* = 0.37 and 0.52, small), RACC (*d* = 1.05 and 1.03, moderate), and RDEC (*d* = 0.94 and 0.95, moderate) than RM and LM. Compared to LM, the RM showed significantly higher RACC (*d* = 0.28, small) and RDEC (*d* = 0.25, small), but lower RHIR (*d* = 0.49, small).

With respect to MD-3, the LM presented significantly higher RHIR (*d* = 0.90, moderate) and RSPD (*d* = 0.56, small) than RM, but RM had significantly higher RTD (*d* = 0.56, small) and RDEC (*d* = 0.35, small) than LM. In addition, the SM displayed significantly lower RTD (*d* = 1.08 and 0.55, moderate and small), RHIR (*d* = 0.58 and 1.38, small and large), and RSPD (*d* = 0.82 and 1.74, moderate and large) compared to RM and LM. However, the SM presented greater RACC (*d* = 0.41 and 0.54, small) and RDEC (*d* = 0.49 and 0.85, small and moderate) compared to RM and LM.

On MD-2, the SM displayed significantly greater RTD (*d* = 0.59 and 0.72, small and moderate), and RSPD (*d* = 1.22 and 1.15, large and moderate) than RM and LM. The SM also exhibited greater RHIR (*d* = 0.40, small) than LM. Finally, in relation to MD-1, the RM showed significantly lower RTD (*d* = 1.13 and 1.10, large) and RACC (*d* = 0.71 and 0.81, moderate) than SM and LM. In addition, RM presented significantly lower RDEC (*d* = 0.75, moderate) than LM.

## DISCUSSION

This study aimed to analyze the effects of microcycle length on external and internal load across training sessions and MD in a professional female soccer team. To the author’s knowledge, this is the first study to examine the influence of microcycle length on workload management in professional female soccer. The main findings of this study were that (a) no differences in external and internal load were found between microcycles of different lengths on MD; (b) for the acquisition days (i.e., MD-4 and MD-3), the highest volume and intensity were found in locomotor and neuromuscular metrics in LM, except for ACC and DEC on MD-3; (c) whereas for MD-2, the highest workload was found for locomotor metrics and internal load metrics in SM, and (d) for MD-1, similar values were reported for all external and internal load metrics regardless of microcycle length.

The load dynamics throughout the conventional competitive microcycle determine that on MD+1, starters typically engage in recovery and compensation sessions for players who participated less than 45–60 minutes in the previous match [4, 13, 15]. Specifically, these compensatory sessions attempt to simulate match demands in order to maintain the player’s physical fitness [15]. In particular, the compensatory training sessions include rondos, high-intensity circuits, and small-sided games (area per player: range from 50 to 75 m^2^) that overload ACC and DEC demands [48–50]. In reference to MD+1, our results showed that TD, ACC, and DEC were higher in SM compared to RM and LM, whereas no significant differences were reported for high-intensity metrics (i.e., HIR and SPD). In the same vein, Oliva-Lozano et al. [26] analyzed the differences in external load according to the length of microcycle in male professional soccer players. Similarly, these authors found that regardless of microcycle length, high-intensity locomotor metrics did not show significant differences between different microcycle lengths at MD+1. Contrary to these results, the ACC and DEC metrics were lower in RM and LM than in SM on MD+1. Perhaps, these differences in mechanical load could be related to longer training session durations in SM (~15 min more than RM and LM).

In contemporary soccer, the mid-week training sessions (i.e., MD-4 and MD-3) presented the highest values of external and internal load throughout the competitive microcycle [13, 15]. Based on previous evidence, the MD-4 was the most demanding session in terms of mechanical load (i.e., ACC and DEC), whilst the MD-3 was more focused on stimulating HSR and sprinting actions [14, 15, 23, 26]. In the current study, the MD-4 showed higher volume and intensity of ACC and DEC, as well as greater RPE and sRPE in RM and LM than in SM. Likewise, the MD-3 exhibited higher volume and intensity in HIR and SPD during RM and LM compared to SM. In this sense, this is the first study to analyze the effect of different microcycle lengths in professional female soccer players, so a direct comparison with previous research is not possible. For instance, Oliva-Lozano et al. [26] reported no significant differences in high-intensity ACC and DEC in male professional soccer players according to microcycle length on MD-4. Consistent with our results, the same study determined that LM presented greater distances covered in HIR than SM on MD-3. This notion is reinforced by previous research which established that mid-week training sessions displayed greater external and internal load during the longest microcycles [27, 28, 32, 51]. Notably, the highest mechanical load in RM and LM could be related to the training contents on MD-4 and the emphasis on optimizing the recovery process 48 hours after the previous match during SM [52]. Specifically, the small-sided games with a small pitch size and a low number of players (area per player: range from 50 to 150 m^2^) were included on MD-4 in RM and LM, contributing to an increase in the number of high-intensity ACC and DEC, while not included in SM for the same day of the microcycle [47, 49]. Regarding MD-3, the SM displayed a lower distance covered in HIR and SPD than the largest microcycles, but female soccer players were exposed to a higher mechanical load (i.e., ACC and DEC). This fact could be related to the need to reorganize training content, combining training drills (i.e., small-sided games and large-sided games) with different load dynamic orientations to prepare the next match strategy and minimize residual fatigue during SM [4, 53].

Previous scientific literature has identified a tapering strategy on MD-2 and MD-1 to reduce fatigue and improve competitive player readiness in professional female soccer [13, 23]. A recent survey on loading patterns and programming practice in elite soccer highlighted that load dynamics on MD-2 change based on microcycle length [4]. In addition, Buchheit et al. [54] found that exposure to near-maximal sprinting speed on MD-2 is associated with a reduction in the likelihood of injury risk in soccer. The current study showed that the SM presented higher values for SPD, RSPD, RPE, sRPE, and maximal speed than RM and LM on MD-2. Conversely, the RM and LM revealed higher values for SPD and maximal speed on MD-3. This is consistent with previous findings showing greater values of HIR and SPD on MD-3 compared to MD-2 during conventional microcycles in professional female soccer players [23, 55]. Of note, the optimal training day for exposure sprint dose is a subject of debate between practitioners and sports scientists in competitive microcycle programming [4, 54]. In relation to the SM structure, Gómez-Díaz et al. [53] stated that a common programming strategy during SM is to integrate two distinct training sessions into a single session. Notably, the MD-2 in the SM structure was characterized by using large-sided games (area per player: > 200 m^2^) that stimulate high-speed activities (i.e., HIR and SPD), and longer training session durations in contrast to RM and LM. These discrepancies could be related to the fact that the SM provides fewer days to prepare tactical match-planning and enhance players’ recovery status. This programming strategy involved structuring the contents of MD-2 in SM similarly to MD-3 in both RM and LM, but with reduced training exposure to minimize potential fatigue effects and promote the player’s match readiness. Therefore, the tapering strategy is more evident during RM and LM than SM due to the management of training loads applied by strength and conditioning coaches. Nevertheless, no differences in physical output were found between microcycles on MD. These results may not align with previous studies, as significant differences between LM, RM, and SM were reported for variables such as ACC, DEC, or HIR, although with small effect sizes [26].

This study has some specific limitations that should be acknowledged when generalizing the current findings. Firstly, the study design is limited to a single professional female soccer team, with microcycle programming decisions made by a specific technical staff. Due to the differences in competitive demands between soccer backgrounds, the findings may not be generalizable to other leagues or competitive standards. Secondly, only one competitive season was analyzed, and it would be interesting to examine data from multiple seasons. Thirdly, the influence of match-related contextual variables (i.e., match outcome, opponent level, or match location) on the training load prescription for the subsequent microcycle was not considered. Additionally, the external load was measured by a GPS system used by several professional clubs, although its validation is limited to some university settings and lacks peer-reviewed studies. However, as previously mentioned, the technical specifications provided by the manufacturer establish high levels of accuracy. It is also important to emphasize that low-cost GPS systems are vital for conducting research in populations with limited financial resources. Lastly, only RPE and sRPE were employed to analyze the internal load demands across training sessions and MD. To address these limitations, further studies should attempt to develop a multi-club and multi-season analysis, model match-related contextual variables effects, use individualized thresholds, and integrate other internal load variables (e.g., heart rate, training impulse, or creatine kinase).

## CONCLUSIONS

Microcycle length is a cornerstone for soccer coaches and sports scientists when programming training sessions, developing recovery strategies, and appropriately managing workloads during competitive microcycles. In this regard, this study demonstrated that female soccer players were exposed to a higher external and internal load during mid-week training sessions (i.e., MD-4 and MD-3) during longer microcycles, except for ACC and DEC on MD-3. However, the SM presented higher values in locomotor metrics than longer microcycles on MD-2. Perhaps, it could be related to the soccer coach’s need to prepare the team for the next match in a shorter time window, combining drills with different load dynamics (i.e., small- and large-sided games) in the same training session.
